# Supplementary material for: Physical Activity during Pregnancy and Newborn Body Composition: A Systematic Review
Source: Int J Environ Res Public Health. 2022 Jun 10;19(12):7127. doi: 10.3390/ijerph19127127 (PMC9222359; doi:10.3390/ijerph19127127)
Supplement: Supplementary file 1 [file ijerph-19-07127-s001.zip › ijerph-1692144-supplementary.pdf]

Table S1. Description of maternal physical activity groups and summary of data reporting.

**Authors, Year, Ref**	**Data Reporting**
Barakat, et al. 2009 [17]	Previously sedentary women randomly assigned to groups, 80 control & 80 exercise (light resistance training)<br>Multiple regressions to study association between maternal body weight with newborn birth weight, birth length, and ponderal index
Bisson, et al. 2015 [18]	Randomly assigned, 25 standard care & 25 supervised (aerobic & resistance) exercise<br>Pre-pregnancy activity plus accelerometry at 14, 28, 36 weeks gestation<br>Repeated measures ANOVA to compare effect of group allocation, time in gestation, & the interaction of both on maternal physical activity levels<br>Neonatal anthropometrics were compared to maternal PA by student t-test & correlation analyses
Clapp et al., 2000 [19]	Women randomly assigned, 22 (8 wks weight bearing) exercise or 24 no exercise<br>Secondary analysis of previously sedentary women between group measures of neonatal outcomes and maternal physical activity
Clapp et al., 2002 [20]	Women randomly assigned to 3 exercise groups organized by intensity 26 (lo-hi) 24 (mod-mod) 25 (hi-lo)<br>Secondary analysis of between group measures of neonatal outcomes and maternal physical activity of previously active women
Seneviratne et al., 2016 [26]	Women randomly assigned, 38 exercise (16 wk home cycling program) & 37 control<br>Regression to evaluate maternal PA on infant birth outcomes<br>Controlled for maternal BMI, ethnicity, parity, & offspring gender
Clark et al., 2019 [21]	Women randomly assigned, 14 aerobic exercise & 22 no exercise<br>Two sample t-tests compare between exercise & control of maternal characteristics<br>Correlations for maternal physical activity, characteristics, and infant outcomes
Garnaes et al., 2017 [22]	Women randomly allocated to 38 supervised exercise (walking & resistance training) or 36 standard of care groups<br>Correlation tests for maternal PA and infant birth weight, birth length, & BMI<br>Controlled for gestational age & parity
Hoffman et al., 2019 [23]	Secondary cohort analysis, women PA behavior during pre-pregnancy & first trimester influenced by education<br>Linear regression used to compare maternal MVPA groups with infant anthropometric outcomes<br>Controlled for pre-pregnancy BMI, parity, & age
Hopkins et al., 2010 [24]	84 women randomly assigned to home-based exercise program or standard of care (control)<br>Repeated measures ANOVA to compare maternal groups and infant body composition
Seneviratne et al., 2017 [25]	Women randomly assigned, 38 exercise & 37 control intervention, grouped by parity<br>Linear regression to analyze maternal BMI & PA effect on infant body composition<br>Controlled for gestational age & ethnicity
Sklempe Kokic et al., 2018 [27]	Women randomly assigned, 20 exercise 22 control (standard care)<br>Comparison of maternal PA with infant body composition
Trak-Fellermeier et al., 2019 [28]	Women randomly assigned, 15 lifestyle intervention & 16 standard of care control<br>Regression analyses to compare maternal PA and infant birth weight, birth length, & Ponderal index
Van Poppel, et al., 2019 [29]	Women randomly assigned to 4 groups of 113 healthy eating, 110 physical activity, 108 both, & 105 standard of care<br>Regression analyses to compare maternal PA and infant body composition

Table S2. Description of maternal physical activity groups and summary of data reporting.

Authors, Year, Ref	Data Reporting
Bisson et al., 2017 [8]	MPA calculated for the 25th, 50th, & 75th percentiles, women were grouped for VPA or no VPA at 17 weeks<br>Maternal PA and infant body composition addressed through multivariate analyses & general linear models
Harrod et al., 2014 [36]	MVPA quartiles calculated<br>Regression analyses to compare maternal PA and infant body composition<br>Controlled for gestational age, offspring sex, gravidity, pre-pregnancy BMI, education, maternal age & race
Collings et al., 2020 [33]	Women grouped into 4 activity levels based on self-report PA<br>Regression analyses to compare maternal PA and infant birth weight, birth length, & body composition
Jones et al., 2020 [37]	MVPA from accelerometer, grouped by quartile<br>Regression analyses to estimate relationship between maternal PA & infant body composition
Diaz et al., 2020 [35]	MVPA from accelerometer, grouped by quartile<br>Regression analyses to estimate relationship between maternal PA & infant body composition
Mudd et al., 2019 [40]	Recall pregnancy PA<br>Regression analyses to compare recalled maternal MVPA and infant body composition<br>Controlled for gestational age, pre-pregnancy BMI, race, breastfeeding, parity, & offspring sex
Rao et al., 2003 [44]	Activity scores from questionnaire classified women into light, moderate, & heavy PA<br>Regression analyses to relationship between maternal PA & infant body composition<br>Adjusted for common/major confounding variables (not specified)
Watson et al., 2018 [45]	Accelerometer MVPA data grouped by quartile<br>Regression analyses to estimate relationship between maternal PA & infant body composition<br>Adjusted for parity, education, & smoking
Nagpal et al., 2018 [41]	Self-reported MVPA, women grouped by active & inactive<br>Correlations between inactive or active group with infant body composition
Badon et al., 2016 [31]	Reported LTPA during pre-pregnancy & early pregnancy, grouped by active or non-active women<br>Regression analyses to estimate relationship between maternal PA & infant body composition
Badon et al., 2018 [30]	Self-reported LTPA (walking & yoga)<br>Regression analyses to estimate relationship between maternal PA & infant body composition
Norris et al., 2017 [42]	Secondary analysis of cohort, self-report data & grouped based on changes in activity at 15 & 20 weeks gestation<br>Regression analyses to estimate relationship between maternal PA & infant body composition
Juhl et al., 2010 [39]	Self-reported physical activity, assigned women to active groups based on types completed<br>Compared pregnancy self-reported PA with infant outcomes to identify association<br>Controlled for parity & offspring sex
Dahly et al., 2018 [34]	Secondary analysis of cohort study<br>Regression used to estimate relationship between maternal lifestyle factors and infant body composition<br>Controlled for pre-pregnancy weight status & gestational weight gain
Przybylowicz et al., 2014 [43]	Physical activity self-report, categorized to sedentary, light, moderate, active<br>Compared reported PA with infant birth weight, birth length, & ponderal index
Joshi et al., 2005 [38]	Women divided by parity<br>Linear regression for physical activity scores, parity groups, & infant body composition outcomes

Table S3. Description of maternal physical activity groups and summary of data reporting.

**Authors, Year, Ref**	**Data Reporting**
Clapp & Capeless, 1990 [46]	77 recreational runners & aerobic dancers with 55 inactive (control)<br>Unpaired Student t-tests, Wilcoxon test, and multiple regression analysis for understanding dose response relationship between exercise performance & infant outcomes
Clapp et al., 1998 [6]	52 women exercised & 52 women were sedentary (control)<br>Between group differences, student t-tests, & Bonferroni correction for multi comparisons between maternal exercise and neonatal outcomes
Tinius, Cahill, Strand, & Cade, 2016 [5]	16 obese active & 16 obese inactive (control)<br>Correlation analyses between maternal leisure activity and neonatal outcomes with partial correlations for potential confounders
